# Capturing the COVID-19 Crisis through Public Health and Social Measures Data Science

**DOI:** 10.1038/s41597-022-01616-8

**Published:** 2022-08-26

**Authors:** Cindy Cheng, Amélie Desvars-Larrive, Bernhard Ebbinghaus, Thomas Hale, Alexandra Howes, Lukas Lehner, Luca Messerschmidt, Angeliki Nika, Steve Penson, Anna Petherick, Hanmeng Xu, Alexander John Zapf, Yuxi Zhang, Sophia Alison Zweig

**Affiliations:** 1 Hochschule für Politik and the TUM School of Social Sciences and Technology at the Technical University of Munich (TUM), Munich, Germany; 2 Unit of Veterinary Public Health and Epidemiology, University of Veterinary Medicine Vienna, Vienna, Austria; 3 Complexity Science Hub Vienna, Vienna, Austria; 4 Department of Social Policy & Intervention, University of Oxford, Oxford, UK; 5 Blavatnik School of Government, University of Oxford, Oxford, United Kingdom; 6 ACAPS, Geneva, Switzerland; 7 Department of Epidemiology, Johns Hopkins Bloomberg School of Public Health, Baltimore, Maryland USA; 8 Department of International Health, Johns Hopkins Bloomberg School of Public Health, Baltimore, Maryland USA

**Keywords:** Politics, Society, Government, Research data, Research management

## Abstract

In response to COVID-19, governments worldwide are implementing public health and social measures (*PHSM*) that substantially impact many areas beyond public health. The new field of *PHSM* data science collects, structures, and disseminates data on *PHSM*; here, we report the main achievements, challenges, and focus areas of this novel field of research.

## Introduction

In response to the COVID-19 pandemic, governments around the world are enacting a wide range of public health and social measures (*PHSM*), also known as non-pharmaceutical interventions, including e.g., school closures, movement restrictions, and contact tracing. These measures not only impose unprecedented restrictions on human behaviour, but have also been implemented at an extraordinary scale, duration and variation. For instance, while many governments have collectively implemented thousands of measures related to lockdowns, curfews, or stay-at-home orders recorded in different datasets, other governments have not imposed any at all (“laissez-faire” strategy)^1^. Meanwhile there has been great variation even among relatively common policies. For example, though most governments implemented lockdown measures which applied to the general population, many governments have introduced their own specifications. Indeed, the Lebanese government limited traffic by allowing citizens with odd or even license plate numbers out on certain days^2^ while in Panama, citizens were allowed out on certain days depending on their sex^3^. In short, *PHSM* have not only shaped the progression of the pandemic but have irrevocably affected how billions of people conduct their lives. Though tracking *PHSM* is key to our understanding of the pandemic’s drivers and impacts, gathering accurate, timely and complete *PHSM* data is a monumental task: government responses to COVID-19 are incredibly varied across time and space and their documentation has been both unstructured and dispersed across a broad range of government and news portals.

Without previous work to guide them, starting from March 2020, more than 40 distinct *PHSM* “trackers” have taken on the challenge of organizing these policies into structured databases that are both understandable to non-experts and available for use in rigorous research. To do so, they have sifted through reams of primary sources, developed structured taxonomies to categorize them, and coordinated tremendous human resources to try to keep pace with the sheer volume of *PHSM* to collect, categorize, clean, and validate. Though *PHSM* trackers are largely associated with the underlying data they process, they should be more holistically understood as research groups (from academia or the public or private sectors) that produce both the taxonomies for describing and understanding government responses to COVID-19 as well as the organizational infrastructures for systematically documenting *PHSM* data in near real-time.

By granting public access to their data, policy trackers in the new field of *PHSM* data science provide an essential foundation for our collective ability to answer pressing questions of interest to researchers, policy-makers and the global community alike including: When and under what conditions are some *PHSM* more or less effective at curbing the spread of the virus? Why do some countries adopt certain *PHSM* while others do not? What unintended political, economic or social consequences have resulted from *PHSM*?

While scientifically rigorous research on these and other questions crucially depends on the availability of timely and high-quality *PHSM* data, the continued provision of this public good is far from guaranteed. As representatives of a consortium of *PHSM* data trackers, raising awareness of the many achievements and contributions of *PHSM* data science over the past two years is of secondary importance to making sure the wider research and policy community is aware of the major challenges that trackers face in sustaining their indispensable work. This review of pressing issues in the field of *PHSM* data science represents a summary of the discussions conducted between over 40 trackers across two *PHSM* conferences hosted on these topics. It further includes the results of 16 survey responses of self-reported data collected and project resources from *PHSM* trackers in our network up until November 2021. For more information on the trackers, see Table 1.

**Table 1:** List of Trackers Analysed in Fig. 1 and that responded to the survey.

Dataset	Survey Participation	Geographic Scope	Active	Last update*	Categories
ACAPS^32^	YES	Worldwide	NO	08.12.20	NPIs, Econ
CCCSL^33^	YES	Worldwide	NO	25.01.21	NPIs
CDC**	NO	Worldwide	NO	28.06.21	NPIs, Econ
CIHI^35^	YES	Canada	YES	25.07.22	NPIs, Vaccines
CMMP^36^	YES	Worldwide	NO	01.08.20	NPIs
COBAP^37^	NO	Worldwide	NO	21.12.21	NPIs
CoronaNet^9^	YES	Worldwide	YES	05.07.22	NPIs, Vaccines
COVID-19 EU Policy Watch^38^	YES	EU+	YES	05.07.22	Econ
COVID-19 Food Trade Policy Tracker^39^	YES	Worldwide	NO	01.06.21	Econ
COVID-19 Health System Response Monitor^40^	YES	Worldwide	YES	05.07.22	NPIs, Econ, Vaccines
Covid-19 Law Lab^27^	YES	Worldwide	YES	05.07.22	NPIs, Vaccines
COVID-19 State Policy Project^41^	NO	USA	NO	09.08.21	NPIs
COVID-AMP^42^	NO	Worldwide	YES	05.07.22	NPIs, Vaccines
HIT-COVID^34^	YES	Worldwide	NO	18.05.21	NPIs
INGSA^43^	NO	Worldwide	NO	11.01.21	NPIs
OxCGRT^8^	YES	Worldwide	YES	05.07.22	NPIs, Econ, Vaccines
Oxford Supertracker^44^	YES	—	YES	—	—
Response2covid19^4^	YES	Worldwide	NO	04.06.21	NPIs, Econ
THF^45^	NO	England	NO	31.12.20	NPIs
NPI B^46^	NO	Brazil	NO	19.10.20	NPIs
UNDP^47^	NO	Worldwide	NO	31.12.21	NPIs, Econ
WHO Euro**	NO	Worldwide	YES	05.07.22	NPIs, Econ, Vaccines
WHO PHSM Dataset^24^	YES	Worldwide	YES	05.07.22	NPIs, Econ, Vaccines
Yale State and Local COVID Restriction Database^48^	YES	USA	YES	05.07.22	NPIs, Econ

*Notes:* In the geographic scope column, EU+ refers to the European Union plus Norway, and USA refers to the United States of America.

In the ‘Categories’ column, NPIs refers to non-pharmaceutical intervention. Since the Oxford Supertracker is a directory of different COVID-19 projects, many of the columns are not applicable to it.

*At the time of writing (July 2022).

**The US Centers for Disease Control and Prevention (CDC) and WHO EURO’s PHSM dataset (WHO Euro) are only publicly available through the WHO PHSM database^24^.

## Major achievements of *PHSM* trackers

**Fig. 1:**
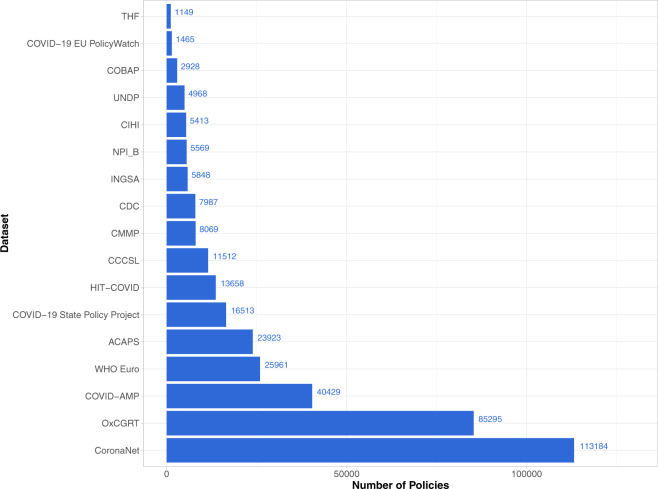
Total number of *PHSM* policies: Collected and curated by *PHSM* databases as of April 2022. For tracker reference, see Table 1.
- Developing novel, structured, and detailed taxonomies customized to capture COVID-19 *PHSM*. To illustrate how much variation there has been in PHSM data structure and taxonomy, we can consider the coding schemes of the two biggest trackers, the OxCGRT^8^ and CoronaNet^9^. While the latter tracker organizes COVID-19 policies in a event database that categorizes policies into over 100 sub-categories with detailed text descriptions, the OxCGRT has a panel structure that categorizes policies along 21 broader policy categories.
- Creating original organisational frameworks and infrastructure to process raw data and information into curated *PHSM* datasets.
- Building significant global networks of data collectors united in the mission to document *PHSM*. They have accumulated considerable experience and knowledge as part of what is arguably one of the largest efforts ever attempted to collect public health data in real time. Across the trackers that filled out the survey, more than 2,000 people have collected data, mostly as student volunteers motivated by the opportunity to contribute to scientific research.
- Making *PHSM* data openly accessible and available in (close to) real time as a public good for researchers, the public, and stakeholders to utilize. Public access to *PHSM* data has played a crucial role in advancing our collective understanding of the countless ways that the pandemic has affected our economies, our communities and our daily lives.
- Fostering international collaboration, coordination, and communication within and between trackers, culminating in two international conference held in 2021. The COVID-19*PHSM*Data Coverage Conference, held in February and March 2021, brought together 40 *PHSM* trackers as well as researchers and *PHSM* data users to share key tracking lessons, identify challenges, and discuss how to enhance pandemic preparedness. Meanwhile the PHSM Research Outcome Conference, held in October 2021, provided an important forum for scholars to share research findings based on *PHSM* data.
- Creating the COVID-19 *PHSM* Network^10^, which represents an important collegial advance in facing current and future challenges raised by the novelty and complexity of collecting *PHSM* data (Fig. 2).

We first provide an overview of what *PHSM* trackers have achieved over the past two years. In particular, they have been:

- Tracking *PHSM* data over time (i.e., from the beginning of the pandemic to present day) and space (i.e., worldwide coverage at both national and subnational levels). To date, trackers have collectively coded more than 365,000 policy responses in their databases (Fig. 1). Because what counts as a policy can differ greatly from dataset to dataset, to maximize comparability, we only included policies that could be represented in an event dataset format where each observation is associated with a unique policy event (as opposed to a panel country-day format where a unique policy event may be captured across multiple rows of observations depending on how long the policy event was in place). Further, note that 365,000 + represents the cumulative number of policies documented by trackers independently; the number of unique policies is likely smaller due to duplication across datasets. To minimize the likelihood of double counting policies, we also only included data from trackers that conducted original data collection (for at least 1000 policies) as opposed reformatting or repurposing existing *PHSM* datasets (e.g., the response2covid19 dataset)^4^. Finally, note that we focus only on groups that have specifically sought to document COVID-19 *PHSM*. Though there have been hundreds of other trackers which have gathered related data on COVID-19, including e.g. public opinion surveys^5^ or data on SARS-CoV-2 infections^6,7^, they are beyond the scope of this commentary. For more information on the trackers covered here, see Table 1.

**Fig. 2:**
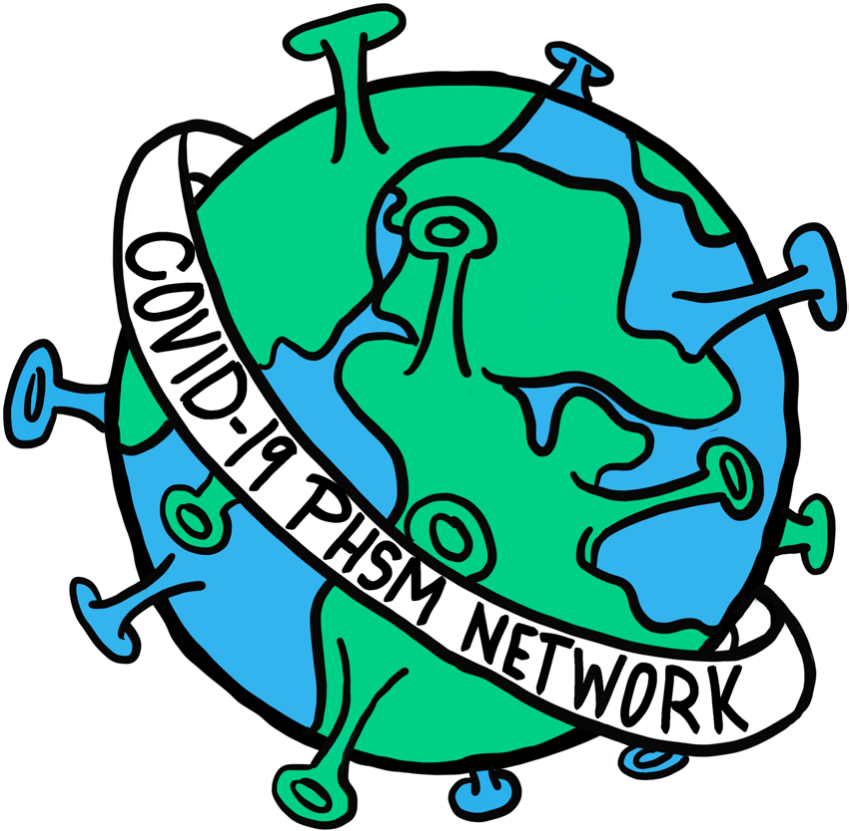
Logo of the newly founded COVID-19 *PHSM* Network. Logo created by Alexandra Williams.

## The value of *PHSM* trackers as essential tools amidst the COVID-19 crisis

*PHSM* trackers are building a foundation for analysing the impact of COVID-19 government policies and interventions. As such they are building remarkable and readily accessible historical records for future generations of scientists, policy experts, and the public to learn from.

To date, combined with multidisciplinary data (e.g., number of COVID-19 cases, deaths, hospitalisations, mobility, and economic data), *PHSM* have provided crucial input for researchers to model and understand the spread of COVID-19. In doing so, they provide an important foundation for evidence-based policymaking and scientific research on the pandemic. For example, data from *PHSM* trackers have been utilized in research to evaluate the impact of *PHSM* on COVID-19 transmission^11–13^, mortality^14^, human rights^15,16^, food prices^17^, health policy and pandemic fatigue^18^. They have also been utilized to describe and explain the cross-country and longitudinal variations in governments’ COVID-19 policy decisions^8,19^. Importantly, *PHSM* data can further be used to communicate accurate scientific knowledge to the public, improve data transparency, hold media outlets accountable for misinterpretation, and avoid misinformation around COVID-19 *PHSM* and their impact as well as their potential consequences.

## Major Challenges in Tracking COVID-19 *PHSM*

*PHSM* trackers have not been able to come by their achievements easily. From developing data taxonomies to building organizational structures for collecting, cleaning and validating data, *PHSM* trackers initiated their efforts without the benefit of precedent. At the beginning of the pandemic, trackers also worked without knowledge of each other’s efforts. While a vast improvement over isolation, greater cooperation among trackers entails its own set of challenges. Our review below provides a holistic overview of both the data and organizational challenges facing *PHSM* trackers individually and as a group. This can be complemented with Shen *et al*.’s^20^ commentary, which provides a more in depth discussion of the various data challenges faced by individual trackers.

### Individual challenges

Data taxonomy forms the basis for comprehensible and meaningful use of *PHSM* data. Each tracker had different strategies for building their taxonomies, and given the peculiarities of how governments implemented COVID-19 *PHSM*, they generally developed them inductively and inferentially. Trackers have found that the main challenge in doing so is developing a standard taxonomy that can both capture the nuances and peculiarities of a given country’s *PHSM* rollout while also allowing for cross-country comparisons. Additionally, ensuring that taxonomies remain relevant over time by including periodic updates (e.g., documenting vaccination policies following the global vaccine rollout) remains an ongoing challenge.

Likewise, data standardization remains a key challenge in *PHSM* data collection (as is true in data collection more broadly). Beyond the enormous variability in definitions of policies and interventions *PHSM* trackers encountered while collecting data from around the globe, lack of data standardization on the national, state/provincial, and local levels represents a major hindrance for data collection^21^. This issue affects not only COVID-19 data but also basic demographic data. Indeed, detailed demographic data is often not available to the public and definitions as well as categories for demographic characteristics vary across countries and states^22^. This disarray not only makes data collection highly challenging but also makes it difficult to compare or identify the multitude of e.g., socioeconomic and health consequences of the pandemic, especially with regards to the most vulnerable populations.

**Fig. 3:**
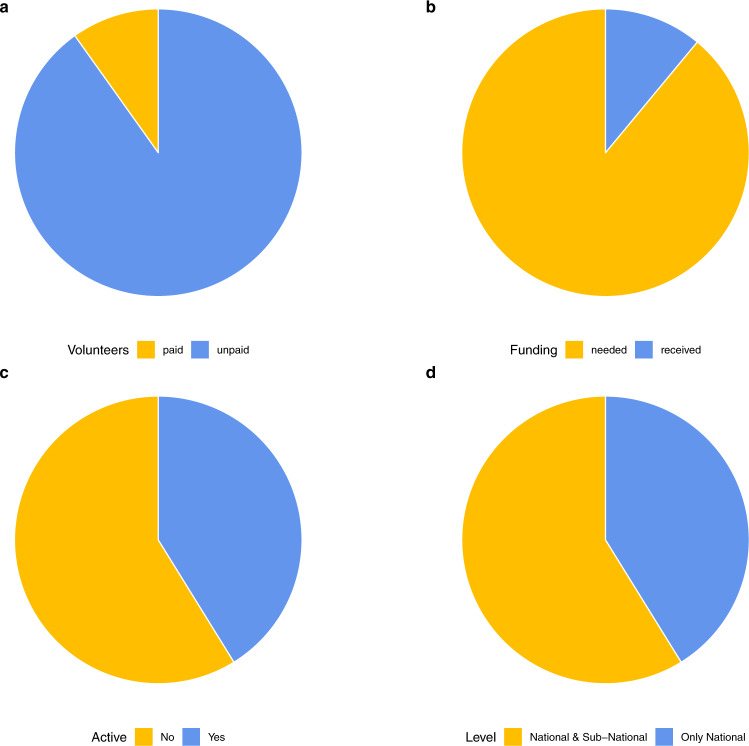
*PHSM* Tracker Survey Responses. Table 1 provides information which trackers participated in the survey. Responses from the tracker survey of *PHSM* Network members to the following questions (**a**) What are the number of paid versus unpaid data collectors? (**b**) What are funding needs compared to received funds? (**c**) Is the tracker still actively coding new policies? (**d**) What governmental level of policies do trackers gather data for?

To collect, clean, and validate this enormous volume of *PHSM* data, most trackers rely on the tremendous contribution of volunteers. However, the corresponding recruitment, training, engagement, and organization of volunteers present enormous challenges. Most volunteers are students and their availability thus fluctuates according to the academic calendar. The reliance on unpaid work also raises questions of research ethics and sustainability. According to our survey, only around 10% of data collectors across are paid; the vast majority are volunteers serving a public good (Fig. 3a).

Many trackers rely on volunteers for data collection not by design, but due to lack of funding. Funding constraints are unfortunately quite severe: many policy trackers have had to stop working because of the lack of continued funding, resulting in wide evidentiary gaps. When trackers do receive funding, it is often short-term because of uncertainty about the pandemic’s duration. According to our tracker survey, only 16% of the overall funding needs by trackers are satisfied (Fig. 3b). This has led to a 65% decline in the number of trackers that are actively collecting data (Fig. 3c). Some trackers have attempted to address this problem by harmonising their data into the few databases with more sustainable funding schemes, which underscores the importance of longer-term funding for sustained *PHSM* data tracking.

### Collective challenges

*PHSM* trackers face challenges not only as individual actors, but also as a collective ecosystem. At the beginning of the pandemic, 40+ *PHSM* tracking projects launched with little to no knowledge of each other due to their emergency nature. These parallel data collection efforts led to the duplication of data, multiple taxonomy strategies across trackers, gaps in data coverage and variation in data quality^23^.

While there is significant data overlap among trackers, many trackers also have unique data coverage in specific domains, such as public health, economic policy, and human rights. Though these differences provide a diversity of perspectives on *PHSM* data tracking, they can lead to difficulties in data utilization. Working toward a single harmonized source might seem like an obvious solution, and indeed the World Health Organization (WHO) has done important work toward this goal^24^. However, this work also underscores the difficulty in data harmonization when underlying data sources are still in the process of being cleaned and organized. More to the point, we believe that there is great value in continuing to maintain diversity in tracking projects. Doing so allows (i) different datasets to be validated against each other (ii) individual datasets to reflect a variety of research priorities and (iii) stakeholders find the dataset that best fits their needs.

The benefits of diversity must be continuously balanced against the costs of data collection, completeness, and quality. With regards to data completeness, *PHSM* trackers have done impressive work in documenting how governments around the world have responded to the pandemic at both national and subnational levels; however, data overlaps and gaps persist. In general, across *PHSM* trackers, data from the “Global North” are overrepresented whereas data from the “Global South” are poor or missing. In the *PHSM* network, only one tracker has its main team physically based in the Global South. Due to funder interests, most data collection is focused on OECD countries and on national policies, leading to large gaps in data collection for less-developed countries and sub-national levels. While over 50% of the bigger trackers collect sub-national data (Fig. 3d), systematic subnational data collection for non-OECD countries is limited to Brazil, China, India, Russia and Nigeria.

With regards to data quality, trackers have learned that local knowledge and/or language skills are essential to gathering complete and accurate information. Because of this, *PHSM* data quality for countries in the Global South is also more likely to suffer because many of the major trackers and their funders are based in the Global North.

Altogether, while all trackers are united in their aim to document government responses to the COVID-19, when considering the sheer number of policies it is possible to collect on the one side, with the diversity of understandings of how to define a policy as well as organisational resources to capture them on the other side, there is a great deal of variation in terms of the scope, quality, and structure of *PHSM* datasets. While providing a definitive guide as to which datasets may be best suited for a given analysis is still premature given the ongoing nature of the pandemic and the attendant data collection thereof, Table 1 provides some broad guidance for adjudicating among different datasets with regards to geographic and temporal dimensions at the time of writing of this commentary.

Ultimately, given the colossal volume and speed of government COVID-19 policy making, greater collaboration between researchers in different fields (e.g., epidemiologists, political scientists, data scientists) as well as communication with policy makers is further needed to understand how to best model and analyse *PHSM* data. Such work would need to start with better integrating *PHSM* them with other relevant COVID-19 data (e.g., COVID-19 cases, deaths and hospitalizations; economic indicators; environmental indicators). In all likelihood, further work would need to be done to develop novel analytical tools for using *PHSM* data to assess the drivers and impacts of the pandemic. While some trackers have made more headway than others on this front (e.g., see Our World in Data’s COVID-19 dashboard: https://ourworldindata.org/coronavirus; or the PERISCOPE COVID Atlas: https://periscopeproject.eu/covid-atlas), the field as a whole still lacks much needed coordination and resources to forward this work.

To address these challenges, in what follows, we outline key focus areas for *PHSM* data science and advocate for greater cooperation and communication among and between *PHSM* trackers.

## Key Focus Areas for Future *PHSM* Data Tracking

In sharing and reflecting on the challenges and lessons learned, we argue that greater emphasis on the following key focus areas will greatly improve our ability to track future *PHSM*:

- Developing a glossary and best practices on *PHSM* data collection, processing, and management.
- Tracking new *PHSM*: Governments continue to implement new *PHSM* as the pandemic progresses (e.g., COVID-19 vaccination policies) and trackers must keep their taxonomies up to date in order to adapt to the changing landscape of COVID-19 *PHSM*.
- Increasing representation of low- and middle-income countries (LMICs) in the Global South: Given the importance of promoting equity in data coverage, we advocate for increased funding of and collaboration with trackers based in and focused on LMICs and the Global South.
- Collecting health equity data: COVID-19 has highlighted and aggravated existing inequities and human rights abuses across the world, such as racism, poverty, and mistreatment of refugees^25,26^. There is an urgent need to collect equity and human rights related data, which is exemplified by several *PHSM* trackers focusing on legal protections, disability justice, and measures of democracy^27–29^.
- Maintaining independent data collection: Protecting the independence, integrity and freedom to pursue research without pressure from governments or funders is vital to ensuring *PHSM* data is free from bias and of the highest quality and accuracy.
- Ensuring that data remain openly accessible: Given the severity of the COVID-19 pandemic, the available technology, and the crucial role that *PHSM* data can play in informing public health policy, making these data open-access advances the common good since they inform policies for the current and future pandemic(s). This is particularly relevant as the burden of the pandemic continues to shift towards lower-income countries with less resources to collect, aggregate, and analyse such data.
- Advocating for greater funding and recognition for volunteers: The availability, quality and timeliness of *PHSM* data is reliant on the thousands of people who have volunteered their time and energy to contribute to this public good. Their efforts deserve not only more recognition but also financial support to sustain tracking efforts going forward.
- Preparing for future pandemics: Given the lack of coordinated *PHSM* data collection at the beginning of the COVID-19 pandemic, we advocate that governments, international organizations, and partners incorporate systematic *PHSM* tracking and analyses as a strategic priority in preparing for the future of this and other pandemic(s). Moreover, the systematic collection of *PHSM* may be valuable in other areas of research, especially in urgent global issues such as climate change, antimicrobial resistance, and social equity. Our practices can serve as a roadmap both for *PHSM* data collection as the COVID-19 pandemic evolves and for future public health emergencies.

## Next Steps and the Importance of International Collaboration

In addressing the focus areas above, greater communication and collaboration will open doors to further input, feedback, and support in order to reach our common goal of providing real-time, high-quality, and complete *PHSM* data to inform the COVID-19 pandemic response. The value of such collaboration was demonstrated during the COVID-19 Data Coverage Conference, at which, together with other participating trackers, we launched the *PHSM* Network and created its underlying mutual framework for building this collaborative ecosystem^10^.

This framework lays an important foundation for future situations in which shared public health challenges call for a collective global response. Thus far, collaboration among individual trackers has significantly increased our shared data collection ability, improved our effectiveness, and helped address the challenges and limitations of *PHSM* tracking. Given that international cooperation among government leaders in response to the COVID-19 pandemic has been inconsistent at best^30^, we hope that the international cooperation we are fostering within the COVID-19 *PHSM* Network can also serve as a model for others seeking to work together in responding to this pandemic^31^.

In order to succeed, however, this network will need the input and assistance of an even wider community–policymakers, donors, and other stakeholders–to provide the necessary feedback and financial support to sustain data tracking efforts given the ever-evolving nature of COVID-19 and the corresponding *PHSM*. The more communication there is among trackers, policymakers, and researchers, the better the quality of the *PHSM* data we can provide by tailoring the data to their information needs.

The longer the pandemic lasts, both the volume and variation of policies are likely to increase, making the provision of complete and high-quality *PHSM* data both within and across countries of immeasurable importance. At the same time however, funding and support for *PHSM* trackers is conversely becoming more limited, threatening the availability, quality, and comprehensiveness of *PHSM* data. In short, the provision of future *PHSM* data is not guaranteed. Our ability to provide *PHSM* data of the desired scope, quality and timeliness to match the importance of *PHSM* data to forwarding scientifically rigorous research is falling short as trackers stop data collection due to lack of funding. While the authors here are responsible for some of the largest *PHSM* trackers currently available, many of them have had to discontinue their work due to funding constraints. ACAPS discontinued new data collection in December 2020^32^; CCCSL discontinued new data collection in April 2021^33^; John Hopkins HIT-COVID discontinued new data collection in May 2021^34^. This not only reduces the diversity of approaches to capturing *PHSM* data but also makes it less likely that future *PHSM* will be documented at all, a possibility which will only grow bigger as the pandemic stretches out further into the future.

Given the complexities of conducting COVID-19 research, it is easy to take for granted the availability of timely, accurate, and high-quality COVID-19 *PHSM* data. While hundreds of trackers and thousands of volunteers have laid the foundation for robust research and evidence-based policymaking, *PHSM* trackers face multiple internal and external challenges in continuing their work. Moreover, to better react to future crises, the infrastructure needs to be developed now to ensure that *PHSM* data collected to document future emergencies can be integrated with other data and analysed as rigorously and efficiently as possible. Greater international collaboration among *PHSM* data trackers as well as more investment and financial support from policymakers and donors is necessary to continue broadening our understanding of the current public health crisis as well as informing future pandemic preparedness efforts.

## Data Availability

All data are available in the main text, Figures and Tables.
